# Multimodal Intelligent Monitoring of Parkinson Disease: Scoping Review of Progress and Translational Challenges

**DOI:** 10.2196/89057

**Published:** 2026-04-17

**Authors:** Jianhui Tan, Xiuzhen Deng, Cuilan Wu, Molin Yao, Jingyi Liao, Haoli Zheng, Changlin Lian

**Affiliations:** 1 The 2nd Clinical Medical College Southern Medical University Guangzhou, Guangdong China; 2 School of Nursing Southern Medical University Guangzhou, Guangdong China; 3 Zhujiang Hospital Southern Medical University Guangzhou, Guangdong China; 4 Guangdong Cardiovascular Institute Guangdong Provincial People’s Hospital Southern Medical University Guangzhou, Guangdong China

**Keywords:** Parkinson disease, multimodal intelligent monitoring, wearable sensors, algorithms, remote monitoring platforms

## Abstract

**Background:**

Parkinson disease (PD) is a progressive neurodegenerative disorder with a rapidly growing global prevalence. Current clinical assessments, such as the Unified Parkinson Disease Rating Scale, are limited by subjectivity and episodic application, creating a need for continuous, objective monitoring solutions. While previous reviews have often focused on single technologies, there is a growing trend toward integrating multiple data sources to provide a more holistic view of PD.

**Objective:**

This scoping review synthesizes progress in multimodal intelligent monitoring systems for PD, focusing on the quantification of motor and nonmotor symptoms, algorithm development, and the clinical translation of remote monitoring platforms. Furthermore, we propose a novel heuristic framework (Care-Platform Transformation in PD [CPT-PD]) that provides a forward-looking conceptual design for integrating these technologies into clinical workflows, demonstrating promising potential for future development.

**Methods:**

A targeted literature search was conducted on August 15, 2025, in PubMed, Web of Science, and China National Knowledge Infrastructure for research published between January 1, 2019, and December 31, 2024. The final search was rerun on January 22, 2026, solely to ensure completeness of coverage for this time window; no articles published after December 31, 2024, were included.

**Results:**

Wearable sensors (n=9) demonstrated high concordance with clinical scores in validation studies (eg, 99% for tremor detection), while computer vision (n=6) achieved moderate agreement with clinician ratings in controlled assessments (intraclass correlation coefficient 0.74 for bradykinesia). For nonmotor symptoms, intelligent systems (n=7) demonstrated sleep disturbance detection with up to 92.9% accuracy and autonomic dysfunction monitoring (n=7) via heart rate variability (area under the curve 0.90) and voice analysis (94.55% accuracy). Algorithm studies (n=16) explored single-modality feature extraction and cross-modal fusion, with emerging applications in federated learning. Remote platforms (n=22) improved medication adherence (172/201, 85.6%) and reduced outpatient visits (by 29% in one study). A heuristic CPT-PD framework was proposed to integrate key components of diagnosis, treatment, and management. Collectively, these advancements demonstrate the technical viability and clinical benefits of shifting from episodic, subjective assessments toward a data-driven, continuous, and multimodal approach to PD management.

**Conclusions:**

While current evidence largely reflects multisensor systems rather than deeply integrated multimodal platforms, the field holds promise for advancing toward genuine data fusion that could further improve clinical decision-making. Persistent challenges include fragmented symptom focus, algorithmic heterogeneity, and barriers to adoption among older adults. Future efforts should build on integrated frameworks such as CPT-PD to develop patient-centered ecosystems, ultimately enabling precision medicine in PD management.

## Introduction

Parkinson disease (PD) is a chronic, progressive neurodegenerative disorder that predominantly affects individuals in middle-to-late adulthood. Classic motor symptoms include bradykinesia, muscular rigidity, tremor, and postural instability, which collectively have a pronounced impact on patient quality of life and the ability to perform daily activities [1]. As the global population continues to age, the prevalence of PD is rising sharply—projections suggest that by 2050, PD will affect 25.2 million people worldwide, representing a 112% increase compared with 2021 [2]. This mounting burden not only impairs individual quality of life but also places escalating pressure on health care systems [3].

Current clinical diagnosis of PD relies heavily on the Unified Parkinson Disease Rating Scale (UPDRS), supplemented by limited laboratory investigations such as magnetic resonance imaging, computed tomography, positron emission tomography, and other modalities for assessing brain metabolism or dopamine synthesis [1]. Nevertheless, these diagnostic approaches have major limitations: they are highly subjective, exhibit substantial interrater variability, and existing methods are insufficient for accurate, real-time monitoring of motor fluctuations in home environments. Consequently, clinicians often lack timely insights into changes in patients’ condition, which hinders optimal adjustment of medication dosages and treatment plans [4]. An urgent need exists for continuous monitoring tools (wearables and smartphones) that can enhance clinical insight while reducing patient burden.

Recent years have witnessed a rapid expansion of intelligent software technologies tailored for chronic disease management. Advances in wearable sensors, the adoption of sophisticated algorithms, and the development of remote monitoring platforms have opened novel avenues for PD assessment. Among these, remote monitoring platforms—integrating diverse sensing modalities (eg, motion, physiological, speech, vision) with advanced algorithms—have emerged as a focal point in research, aiming to bridge gaps in traditional evaluation methods and support precise, home-based monitoring.

It is important to clarify that, although the term “multimodal” conceptually implies deep algorithmic fusion of heterogeneous data streams, the current technological landscape for PD monitoring largely consists of multisensor systems that collect data from multiple sources but analyze them in parallel or with limited integration. Throughout this review, we use “multimodal intelligent monitoring” as a broad descriptor encompassing this spectrum, and we explicitly indicate when a system achieves genuine multimodal fusion versus when it operates at a multisensor level.

This review focuses on progress in remote monitoring platforms and the management of PD from 2019 to 2024, with an emphasis on 3 primary domains:

- Clinical performance of wearable sensors: assessment of their utility and patient compliance in quantifying PD symptoms, including both motor and nonmotor manifestations.
- Algorithmic approaches: analysis of unimodal and cross-modal feature extraction strategies, evaluating efficiency and persisting challenges.
- Clinical translation of remote monitoring platforms: appraisal of current integration status, clinical outcomes, and translational obstacles.

By systematically reviewing advances and challenges in these areas, this review aims to inform future optimization and successful clinical adoption of closed-loop PD management systems, emphasizing a comprehensive “diagnosis-treatment-management” approach.

## Methods

### Search Strategy and Study Identification

This review employed a targeted, purposive sampling method for the literature search rather than undertaking a fully exhaustive systematic review. This strategy aimed to identify representative, high-impact research published between 2019 and 2024 that documents essential breakthroughs and persistent hurdles in remote monitoring platforms for PD. The methodological design and reporting conformed to the PRISMA-ScR (Preferred Reporting Items for Systematic Reviews and Meta-Analyses Extension for Scoping Reviews; Multimedia Appendix 1) extension guidelines, thereby ensuring transparency and rigor throughout the review process [5]. A comprehensive literature search was initially conducted on August 15, 2025, and then rerun on January 22, 2026.

### Key Term Definitions

To ensure conceptual clarity and facilitate understanding for readers from diverse backgrounds (including clinicians), we define the key technical terms used throughout this review in Table 1. Readers are encouraged to refer to this table when encountering these terms in the following sections.

**Table 1:** Definitions of the term “multimodal intelligent monitoring,” “remote monitoring platform,” and “(platform) integration.”

Key terms	Definition
Multimodal intelligent monitoring	Systems that collect and analyze data from at least two sensing modalities (eg, motion, speech, or vision) for Parkinson disease assessment. Approaches range from parallel data analysis in multisensor setups to advanced joint algorithmic integration, although true multimodal fusion remains challenging. Throughout this review, we use the umbrella term “multimodal intelligent monitoring” to encompass the full spectrum from basic multisensor data collection to advanced algorithmic integration, while explicitly noting the level of fusion achieved by specific systems when evidence permits.
Remote monitoring platform	An integrated software-hardware system enabling continuous, autonomous collection, transmission, and processing of patient data outside clinical settings (primarily at home). It delivers actionable insights to health care providers, patients, or both, to support clinical decision-making, with continuous monitoring as its core function.
(Platform) integration	The seamless interfacing and synergistic operation of diverse data sources, algorithms, user interfaces, and clinical workflows within a remote monitoring platform, creating a cohesive system with greater combined utility than the sum of its parts.
Wearable sensor	A body-worn device (eg, wristwatch, patch, insole) incorporating inertial measurement units or physiological sensors to continuously capture motor and nonmotor signals in free-living environments.
Computer vision	An artificial intelligence field that employs cameras and deep learning algorithms (eg, pose estimation) to quantitatively analyze human movement and behavior from video, enabling contactless assessment of motor symptoms.
Digital biomarker	A physiological or behavioral indicator collected via digital tools (eg, sensors, smartphones), such as gait speed or tremor frequency, used to measure disease presence, severity, or progression.

### Search Strategy

We conducted a comprehensive literature search in accordance with the PRISMA-S (Preferred Reporting Items for Systematic Reviews and Meta-Analyses Literature Search Extension) guidelines to ensure transparent and reproducible reporting [6]. We searched 3 electronic databases—PubMed (National Center for Biotechnology Information website), Web of Science Core Collection, and CNKI (China National Knowledge Infrastructure)—from January 1, 2019, to December 31, 2024. The searches were conducted initially on August 15, 2025, and updated on January 22, 2026. It is important to note that the updated search in 2026 was performed solely to verify the completeness of the 2019–2024 period; no articles published after December 31, 2024, were included in this review. We performed the search in each database individually, adapting the search strings accordingly.

To maximize sensitivity and precision, we developed a structured search strategy based on 4 core concept groups:

- Population: PD.
- Sensing modalities: wearable, inertial measurement unit (IMU), computer vision, speech.
- Intelligent methods: machine learning, deep learning, algorithms, multimodal fusion, digital biomarker.
- Platform level: remote monitoring, telemedicine, mobile health (mHealth), digital health.

Synonyms and related terms within each group were combined using the OR operator, and the 4 groups were then combined using AND.

Additionally, the 3-step search strategy recommended by the Joanna Briggs Institute for scoping reviews was employed to maximize sensitivity and comprehensiveness while balancing feasibility [7]. We used phrase searching (with quotation marks) and other precision techniques to ensure accurate retrieval. The full search strategies for all databases, including the exact search strings, filters, and limits used, are provided in Multimedia Appendix 2. For CNKI, the search terms were carefully translated from English, and the search was limited to peer-reviewed journal articles.

We used CiteSpace 6.3.R1 to remove duplicate records identified across the different databases after the initial retrieval. The primary selection of studies was then performed through manual screening of titles, abstracts, and full texts to identify representative and landmark studies that document essential breakthroughs and persistent hurdles in remote monitoring platforms for PD. In addition to database searching, we performed citation searching (snowballing) by manually reviewing the reference lists of all included studies and relevant key reviews to identify additional eligible articles [8].

We did not contact study authors or subject experts to identify unpublished or additional studies. The search strategy was not formally peer reviewed by an information specialist or librarian.

### Eligibility Criteria

#### Study Type

(1) Eligible studies include original research involving human participants (such as randomized controlled trials, cohort studies, case-control studies, cross-sectional studies) or comprehensive reviews (such as systematic reviews and scoping reviews). (2) Studies must be published between January 1, 2019, and December 31, 2024. (3) Only publications in English or Chinese are considered. (4) All included literature must be peer-reviewed, formal publications.

#### Participants

The study population must consist of patients with clinically diagnosed PD, with no restrictions on age, disease stage, or treatment background.

#### Core Concept

Studies must closely revolve around the theme of “monitoring technologies for Parkinson disease.” Specifically, they should report data or provide in-depth discussion on at least one of the following aspects: (1) *technological performance,* such as the accuracy, sensitivity, specificity, reliability, or feasibility of the monitoring technology; (2) *clinical validation,* such as the effectiveness of the technology in real-world or clinical settings, its correlation with clinical assessment outcomes, or its impact on the clinical decision-making process; and (3) *implementation challenges,* such as barriers, limitations, cost, accessibility, or ethical issues encountered during the practical promotion and application of the technology.

#### Context

This study does not impose restrictions on the application settings of the technologies (eg, home, community, clinic, hospital) or on geographic or cultural backgrounds, aiming to comprehensively gather relevant evidence from a global perspective.

### Exclusion Criteria

(1) Non–peer-reviewed publications, including editorials, commentaries, letters, conference abstracts (unless they provide sufficiently detailed technical data), and preprints; (2) studies limited to animal models, in vitro experiments (cell studies), or primarily focused on neurodegenerative diseases other than PD; (3) publications not specifically centered on PD monitoring or those that mention the topic only incidentally; and (4) duplicate publications or instances in which the full text is unavailable.

### Study Selection and Data Extraction

**Figure 1:**
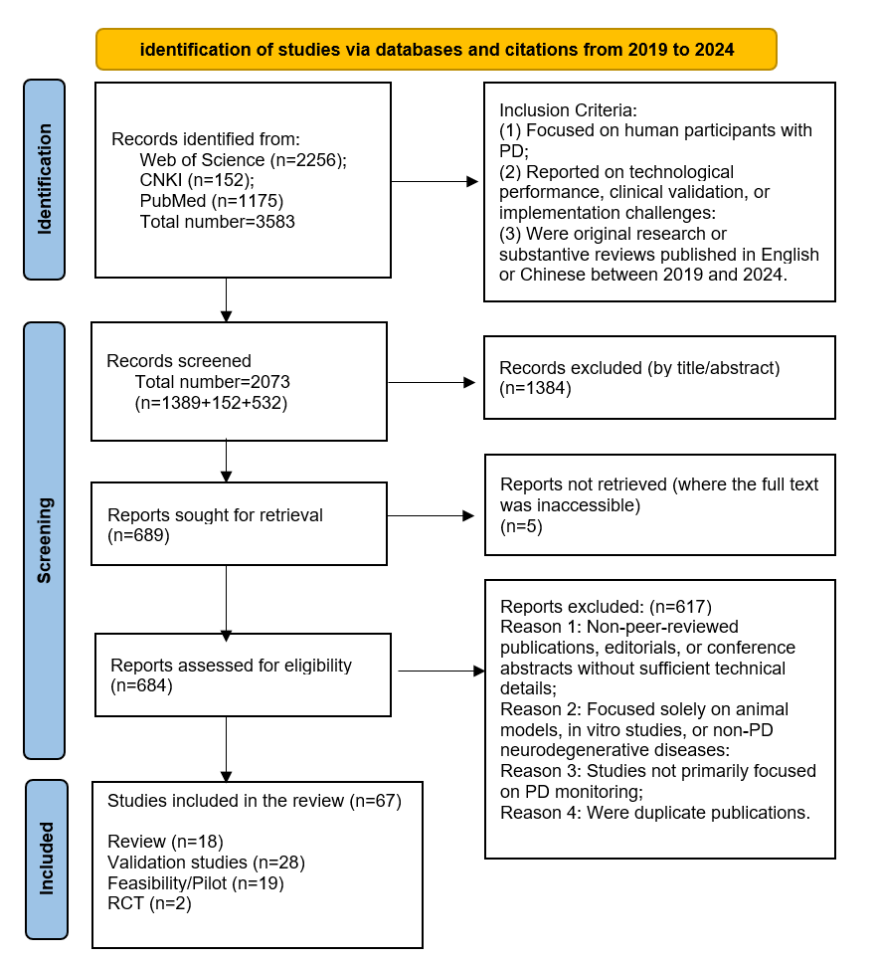
PRISMA-ScR (Preferred Reporting Items for Systematic Reviews and Meta-Analyses Extension for Scoping Reviews) flow diagram of the study selection process for a scoping review of multimodal intelligent monitoring systems in Parkinson disease (2019-2024). PD: Parkinson disease; RCT: randomized controlled trial.

Duplicates were eliminated using CiteSpace 6.3.R1, after which 2 independent reviewers (JHT and XZD) screened article titles and abstracts for relevance. The full texts of potentially eligible studies were then evaluated, with any disagreements resolved by consensus or by involving a third reviewer (CLW). The study selection workflow is outlined in a PRISMA-ScR flow diagram (Figure 1).

### Analytical Framework and Synthesis

This review utilized an a priori analytical framework organized into 3 key technological domains: (1) Symptom Assessment (Hardware Systems); (2) Algorithmic Implementation (Software Methods); and (3) Remote Monitoring Platforms (Integrated Systems).

Studies meeting the eligibility criteria were thematically classified into the respective technological domains to facilitate structured synthesis. For each study, relevant data were extracted concerning technological features, performance metrics, and identified limitations.

### Limitations of Method

This synthesis does not incorporate risk-of-bias assessments or evidence grading due to its nature as a scoping review rather than a full systematic review. The heterogeneity observed in study metrics, technological modalities, and patient populations precluded the conduct of meta-analyses. Nevertheless, this evidence mapping helps identify methodological gaps for future regulatory-compliant research.

Substantial clinical and methodological diversity was identified among the included studies, precluding direct meta-analysis and limiting the comparability of performance results. As a formal risk-of-bias evaluation was not performed, the effect of study quality on reported outcomes may remain a confounding factor and should be interpreted cautiously.

## Results

All search results were exported and managed using EndNote reference management software (Clarivate Plc). The number of records identified from each source and the selection process are reported in the PRISMA (Preferred Reporting Items for Systematic Reviews and Meta-Analyses) flow diagram (Figure 1).

The selection flowchart summarizing the included studies is depicted in Figure 1. A total of 67 studies met the eligibility criteria and were included in this scoping review. Details are provided in Multimedia Appendix 3. Of these, 49 were original research articles and 18 were reviews. Tables 2 and 3 maps the 49 original studies across study design, technology modality, symptom focus, and study maturity, providing a visual gap map of the current primary evidence. Insights from the 18 reviews are integrated throughout the thematic sections below to contextualize the findings and highlight persistent challenges.

**Table 2:** Characteristics of included original studies: study type, technological modalities, and symptom domains.

References	Study type					Technology modality						Symptom domain				
	Technical validation	Clinical validation	Feasibility/pilot	RCT^a^	Wearable-Inertial measurement unit		Wearable-physiological	Computer vision	Speech analysis	Multimodal fusion	Motor		Sleep	Autonomic	Neuropsychiatric	Speech
Joshi et al [9]		✓			✓						✓					
Antonini et al [10]		✓			✓						✓					
Cabo-Lopez et al [11]			✓	✓	✓						✓					
Isaacson et al [12]			✓	✓	✓						✓					
Farid et al [13]	✓	✓			✓					✓	✓					
Botros et al [14]	✓		✓		✓					✓	✓					
Morinan et al [15]	✓	✓						✓			✓					
Chien et al [16]	✓	✓						✓			✓					
Yang et al [17]	✓	✓			✓					✓	✓		✓	✓		
Sigcha et al [18]	✓	✓	✓		✓						✓					
San-Segundo et al [19]	✓	✓	✓		✓						✓					
Borzì et al [20]	✓				✓						✓					
Amato et al [21]	✓								✓							✓
Tsakanikas et al [22]	✓				✓						✓					
Ko et al [23]	✓				✓		✓						✓			
Skibińska and Hosek [24]	✓							✓	✓	✓	✓					✓
Moore et al [25]	✓		✓		✓			✓		✓	✓					
Slemenšek et al [26]	✓				✓		✓			✓	✓					
Lipsmeier et al [27]		✓			✓				✓	✓	✓				✓	✓
Shin et al [28]	✓	✓					✓		✓	✓						✓
Shen et al [29]	✓				✓						✓					
Madrid-Navarro et al [30]	✓		✓		✓		✓			✓			✓			
Wu and Cronin-Golomb [31]	✓		✓		✓					✓			✓		✓	
Mirelman et al [32]	✓		✓		✓					✓			✓		✓	
Schalkamp et al [33]		✓			✓		✓			✓			✓	✓	✓	
Gnarra et al [34]	✓		✓		✓		✓			✓	✓		✓			
Oz et al [35]	✓	✓					✓			✓			✓			
van Wamelen et al [36]		✓			✓						✓			✓	✓	
Raschellà et al [37]	✓	✓	✓		✓					✓			✓			
Obayashi et al [38]		✓					✓						✓			
Lavorgna et al [39]			✓	✓	✓		✓			✓	✓		✓		✓	
Suzuki et al [40]	✓	✓			✓		✓							✓		
Almeida et al [41]	✓								✓							✓
Carrón et al [42]	✓		✓						✓							✓
Polverino et al [43]			✓				✓							✓		
Lim et al [44]	✓	✓						✓	✓	✓	✓					✓
Kyritsis et al [45]	✓	✓			✓						✓					
Silburn et al [46]	✓	✓	✓								✓					
Alves et al [47]		✓	✓								✓				✓	
Thankathuraipandian et al [48]	✓	✓	✓		✓		✓				✓				✓	
Hoffman et al [49]	✓				✓		✓				✓				✓	
Múrias Lopes et al [50]	✓	✓			✓						✓					
Ileșan et al [51]	✓				✓						✓					
Wijers et al [52]		✓														
Tarousi et al [53]	✓				✓			✓			✓					
Katie and Nicky [54]	✓				✓					✓	✓					
Gatsios et al [55]			✓		✓						✓					
Fabbri et al [56]			✓							✓						
Song et al [57]	✓								✓							✓

^a^RCT: randomized controlled trial.

**Table 3:** Characteristics of included original studies: study maturity and sample size.

References	Study maturity						Sample size			
	Lab/controlled	Short-term home	Long-term home	Multicenter validation	Clinically available	Small (<30)		Medium (30-100)	Large (>100)	Multicenter (>200)
Joshi et al [9]				✓	✓			✓		
Antonini et al [10]				✓	✓			✓		
Cabo-Lopez et al [11]		✓			✓			✓		
Isaacson et al [12]		✓			✓	✓				
Farid et al [13]	✓				✓	✓				
Botros et al [14]		✓				✓				
Morinan et al [15]				✓						✓
Chien et al [16]	✓							✓		
Yang et al [17]			✓	✓						
Sigcha et al [18]	✓					✓				
San-Segundo et al [19]	✓	✓				✓				
Borzì et al [20]		✓						✓		
Amato et al [21]	✓							✓		
Tsakanikas et al [22]	✓					✓				
Ko et al [23]		✓						✓		
Skibińska and Hosek [24]	✓								✓	
Moore et al [25]	✓	✓				✓				
Slemenšek et al [26]	✓					✓				
Lipsmeier et al [27]		✓		✓						✓
Shin et al [28]	✓	✓		✓				✓		
Shen et al [29]		✓						✓		
Madrid-Navarro et al [30]	✓	✓						✓		
Wu and Cronin-Golomb [31]		✓				✓				
Mirelman et al [32]		✓				✓				
Schalkamp et al [33]			✓						✓	
Gnarra et al [34]		✓				✓				
Oz et al [35]	✓	✓						✓		
van Wamelen et al [36]		✓							✓	
Raschellà et al [37]	✓	✓				✓				
Obayashi et al [38]		✓							✓	
Lavorgna et al [39]			✓					✓		
Suzuki et al [40]	✓					✓				
Almeida et al [41]	✓							✓		
Carrón et al [42]	✓	✓						✓		
Polverino et al [43]		✓				✓				
Lim et al [44]	✓								✓	
Kyritsis et al [45]	✓	✓	✓			✓				
Silburn et al [46]	✓				✓	✓				
Alves et al [47]	✓				✓	✓				
Thankathuraipandian et al [48]		✓						✓		
Hoffman et al [49]		✓				✓				
Múrias Lopes et al [50]	✓				✓				✓	
Ileșan et al [51]	✓					✓				
Wijers et al [52]			✓		✓				✓	
Tarousi et al [53]		✓				✓				
Katie and Nicky [54]	✓									✓
Gatsios et al [55]		✓						✓		
Fabbri et al [56]										
Song et al [57]	✓								✓	

The following sections report various performance metrics (eg, accuracy, sensitivity, area under the curve [AUC], intraclass correlation coefficient [ICC], adherence rates) derived from the included studies. It is important to emphasize that these metrics are highly context-dependent. They reflect performance within the specific study designs, participant cohorts, validation protocols, and technological setups from which they originate. Direct numerical comparisons across different studies, technologies, or symptom domains are not appropriate and may be misleading. These figures are presented to illustrate the current state of evidence and technological potential within each domain, not to establish a definitive hierarchy of performance or clinical readiness.

### Digital Evaluation of PD Motor Symptoms

#### Wearable Sensors

Wearable sensors, especially devices utilizing IMUs, have markedly improved the objectivity and continuity of home-based monitoring for PD motor symptoms through multisite deployment and functional integration (Multimedia Appendix 3). Wearable systems employ either distributed placement (eg, wrist, ankle, waist) or targeted designs, enabling concurrent monitoring of core motor features such as tremor, bradykinesia, and gait abnormalities, as well as quantitative tracking of motor fluctuations and dyskinesia [9-12]. Multisensor solutions—such as PDMonitor and Kinesia 360—enhance symptom detection sensitivity by simultaneously monitoring the limbs and trunk, while single-device platforms, such as Personal KinetiGraph (PKG) smartwatches and STAT-ON, prioritize efficient identification of specific symptom domains [9-11,13].

Recent innovations in sensor design extend beyond conventional motion signal detection. For instance, smart insoles such as FeetMe Monitor deliver multidimensional analysis of gait and plantar pressure by merging IMU and pressure sensor data [13]. However, clinical implementation of multisensor technologies is impeded by concerns regarding wearability and a tendency toward lower long-term adherence as the number of sensors increases. Small-scale studies report high adherence (13-16 hours/day) with 3 or fewer sensors [14], although this may not generalize beyond motivated populations. Increased sensor burden is linked to discomfort and practical obstacles (eg, difficulty dressing), elevating dropout rates in less controlled and longer-term deployments. Furthermore, older patients frequently report usability challenges, leading to lower acceptance of complex multisensor systems [58].

Beyond initial device costs, the sustainability of mobile PD monitoring technologies poses an economic challenge. A review by the International Parkinson and Movement Disorder Society Task Force on Technology emphasizes the current lack of robust business models to incentivize health care payer reimbursement, coupled with a dearth of cost-effectiveness analyses [59]. Hidden costs such as maintenance, data subscriptions, and clinician interpretation time compound the economic burden and create significant barriers for resource-limited populations and health care systems. This highlights the urgent need for simplified, affordable monitoring solutions.

The clinical translation of emerging sensor modalities (eg, sweat analyzers, smart insoles) is hampered by data standardization challenges: (1) the absence of cross-manufacturer calibration standards impedes direct comparison of metrics such as gait symmetry or plantar pressure across devices (eg, FeetMe vs pressure mats); (2) temporal alignment difficulties in fusing heterogeneous sensor streams (such as synchronizing IMU and biochemical data) complicate reliable multimodal analysis and commonly require sophisticated hardware or software solutions [58].

These advances improve symptom quantification and treatment planning. Bio-integrated remote monitoring platforms with flexible, stretchable electronics are particularly promising for overcoming wearability constraints. By forming comfortable, skin-conformal interfaces, these next-generation systems minimize motion artifacts and enhance patient acceptance, representing a crucial advance for sustained long-term monitoring [60].

The diverse landscape of wearable technologies, as summarized in Table S1 in Multimedia Appendix 3, demonstrates a common pursuit of high objective performance (eg, accuracy and specificity often >0.85 for PDMonitor). However, direct comparison of these metrics across devices is challenging due to pronounced methodological heterogeneity. Studies employed different gold standards (eg, UPDRS items, Abnormal Involuntary Movement Scale, expert diary), patient cohorts (varying disease stages), and outcome definitions (eg, thresholds for symptom detection). For instance, although PKG data led to treatment changes in most visits (71/85, 84%), its symptom detection profile (bradykinesia, 15/30, 50%, and 10/30, 33%, dyskinesia) reflects its specific algorithmic focus and validation context, not necessarily an absolute performance ranking. This heterogeneity indicates that high accuracy figures represent proof of efficacy within specific study frameworks, highlighting potential rather than universal clinical readiness. Key translational gaps, consistent across devices, include the need for standardized validation protocols and real-world cost-effectiveness analyses.

#### Computer Vision Technology

The adoption of computer vision technology combined with algorithms has introduced a powerful approach for remote, quantitative assessment of PD motor symptoms. Applications encompass diverse domains, from analyzing respiratory patterns to evaluating total body movements. Deep learning and pose estimation algorithms (eg, MediaPipe, OpenPose) enable automated measurement of indicators such as finger-tapping amplitude, hand speed, and limb agility via standard cameras or depth sensors. Notable achievements include multicenter validations of OpenPose, which, under controlled assessment protocols, obtained over 80% binary classification accuracy for leg movements and moderate concordance with clinician ratings (ICC 0.74) [15]. ICC over 0.7, while encouraging for research, falls short of the standard required (ie, >0.9) for clinical replacement, limiting these tools to adjunctive roles. Similarly, analyses indicate that computational metrics can be statistically correlated with Movement Disorder Society-UPDRS (MDS-UPDRS) scores (eg, ρ=0.361, *P*<0.1) [16], yet this magnitude accounts for only about 13% of clinical variance, suggesting that current computer vision approaches have yet to fully capture the spectrum of symptom severity as evaluated clinically. In parallel, novel approaches such as Massachusetts Institute of Technology’s respiratory analysis system leverage long short-term memory models to interpret nocturnal breathing for disease severity prediction, achieving an AUC of 0.89 in their validation cohort, indicating strong discriminative ability in a research context. However, as with all laboratory-derived metrics, its performance in unselected real-world populations remains to be validated [17]. These advances promote objective, contactless evaluation and facilitate home-based monitoring and remote care.

Nonetheless, the deployment of computer vision in uncontrolled home environments faces robustness limitations. The accuracy of skeletal tracking algorithms is sensitive to nonoptimal conditions: subpar lighting, occlusion, and camera angles can diminish performance. Comprehensive reviews confirm that both camera parameters and environmental factors critically affect pose estimation accuracy [61] and that current markerless systems have strict requirements for acquisition environments [62]. Enhancing robustness for home use requires strategies such as data augmentation (simulating varying lighting and backgrounds), expanding training datasets to include multiple scenarios, and employing low-light imaging hardware and algorithms.

Additionally, the use of video-based assessments raises explicit patient privacy concerns. Video data—including facial and bodily motion—constitute biometric identifiers and are protected health information under the Health Insurance Portability and Accountability Act (HIPAA). Compliant systems must employ comprehensive safeguards: technical measures (encryption, anonymization), administrative protocols (business associate agreements and strict access controls), and physical security (secured acquisition and storage devices). For example, deidentifying video data before analysis aligns with HIPAA’s Privacy Rule and is foundational to privacy-preserving clinical practice [63].

The current reliance on standardized laboratory tasks, while facilitating initial validation, restricts ecological validity and scalability. A future priority is capturing spontaneous, real-world motor symptoms in unstructured daily settings, as this represents a critical next step for computer vision to achieve continuous and clinically meaningful PD assessment.

A comparative view of computer vision systems (see Table S2 in Multimedia Appendix 3) reveals a trend toward robust performance in controlled settings (eg, AUCs of 0.85-0.91 for the MIT respiratory system). However, a critical appraisal must consider the validation context. High ICC values (eg, 0.74 for KELVIN) and correlations with UPDRS, although statistically significant, account for only a limited portion of clinical variance and originate from protocols using standardized, scripted movements. This indicates a current limitation in ecological validity—the ability to assess spontaneous symptoms in naturalistic environments. The performance decline observed in some applications when moving from controlled to uncontrolled settings further illustrates this “bench-to-bedside” gap. For example, one study reported that voice analysis accuracy decreased from 92% in laboratory conditions to 71% in home environments—a 21-percentage-point decline that highlights the vulnerability of laboratory-trained models to real-world environmental variability (background noise, inconsistent recording conditions). This finding underscores that high accuracy in controlled studies does not guarantee clinical utility in home settings. Therefore, the metrics in Table S2 in Multimedia Appendix 3 primarily serve as benchmarks for technical validation under optimal conditions. The next developmental leap requires demonstrating that these systems can maintain accuracy, robustness to environmental variables (eg, lighting, occlusion), and user-friendliness during continuous, unstructured home monitoring, which remains a significant translational challenge.

### Intelligent Recognition of PD Nonmotor Symptoms

Nonmotor symptoms, including sleep disturbances, autonomic dysfunction, speech impairment, mood disorders, and sensory abnormalities, affect the majority of patients with PD and often precede the onset of classical motor symptoms. These manifestations substantially impair quality of life but are frequently underrecognized in routine clinical assessments due to their subjective nature and episodic evaluation. Intelligent identification technologies, therefore, offer particular value by enabling continuous, objective, and ecologically valid monitoring of nonmotor symptom fluctuations.

In this section, studies are categorized according to sensing modalities, including single-modality and multimodal approaches, without implying uniform levels of data fusion or integration.

The cited accuracy and AUC values, while promising, are predominantly derived from controlled pilot studies or validation cohorts with specific inclusion criteria. Their generalizability to broader, unselected PD populations and home environments requires further confirmation.

#### Intelligent Recognition of Sleep Disturbances

Sleep disturbances are widely prevalent in PD. Monitoring technologies range from single-motion recorders to multimodal systems. Flexible electrode arrays combining electroencephalogram, electrooculogram, and electromyography signals have achieved strong agreement with the gold-standard laboratory video polysomnography, demonstrating a sensitivity of 76.8% for detecting muscle atonia during rapid eye movement sleep [35]. Wrist-worn actigraphy devices, using triaxial accelerometry, have delivered up to 92.9% screening accuracy for rapid eye movement sleep behavior disorder in laboratory settings; during 2-week home monitoring, models achieved 100% accuracy within specific PD subgroups and 94.4% accuracy among controls with insomnia [37]. However, the generalizability of these findings requires substantiation through larger, multicenter cohorts.

The Kronowise 3.0 device, integrating wrist temperature, acceleration, and environmental light exposure, reveals no significant discrepancies in core sleep parameters when compared with video polysomnography [30]. Multimodal signal technologies enhance both accuracy and convenience, particularly in home environments, thereby supporting early detection and screening of sleep disturbances in PD.

Environmental factors also significantly influence sleep in PD, as verified by interventional studies. Insufficient daylight exposure and excessive nocturnal light have a direct negative impact on sleep efficiency among patients [38]. Concurrently, a decreased frequency of nighttime bed-turning—confirmed by waist-worn accelerometers—correlates with worsening motor symptoms [64]. Integrated smartphone and wearable trackers have identified a bidirectional causal link between poor sleep quality and subsequent daytime anxiety levels [31], and sleep headbands demonstrate that longer supine sleep periods correspond with heightened dyskinesia severity [34]. Collectively, these technologies provide valuable insights for clinical management.

Despite these advancements, monitoring performance often declines when conducted in real-life home environments due to environmental noise, variability in sensor placement, and inconsistent user compliance. This indicates that findings from controlled settings may not readily generalize.

In addition, systems based on wearable devices have limited sensitivity in detecting lighter sleep stages, particularly transitional states such as N1 sleep. Most reported performance indicators are derived from pilot groups with specific inclusion criteria and should therefore be interpreted with caution. Overall, the existing evidence supports the feasibility of intelligent sleep monitoring in the management of PD, but its reliability and generalizability in long-term, free-living environments still require further confirmation.

#### Intelligent Recognition of Autonomic Dysfunction

Autonomic nervous system dysfunction in PD affects cardiovascular, gastrointestinal, and thermoregulatory domains. Intelligent assessment technologies directly capture physiological signals (Table S4 in Multimedia Appendix 3).

In cardiovascular monitoring, chest straps (eg, POLAR H10) and wristwatches (eg, POLAR V800) facilitate 24-hour heart rate interval recording. Heart rate variability metrics such as SD of normal-to-normal intervals and coefficient of variation of R-R intervals demonstrate high diagnostic utility (AUC 0.90) and can be detected before overt clinical symptoms [40]. Wireless blood pressure monitors offer simultaneous measurement of blood pressure, heart rate, oxygen saturation, and body temperature, autonomously detecting and signaling episodes of orthostatic hypotension with intelligent alerts and support for teleconsultations [43].

In auditory symptom detection, professional and smartphone microphones have demonstrated impressive accuracy rates of 94.55% and 92.94%, respectively, for PD voice analysis [41]. Mobile voice analytics systems also show efficacy in supporting symptom identification in controlled settings [42].

Multimodal intelligence is key for early detection. Combined biometric analysis using smartphone cameras and voice signals from laboratory-controlled samples allows joint assessment of facial expression and voice, aiding early-stage PD diagnosis (AUC 0.85); however, broader clinical validation is needed. Smartwatches can monitor swallowing dysfunction by detecting delayed hand-to-mouth movements [45], while PKG recorders link bradykinesia to constipation, providing quantifiable gastrointestinal dysfunction metrics [36].

Although wearable systems can correlate sleep activity with autonomic function scores, personalized prediction remains challenging [33]. Interpretation of metrics such as heart rate variability is complicated by inter- and intraindividual variability, particularly during longitudinal monitoring. Addressing these challenges requires the establishment of personalized reference baselines and advanced analytical techniques (eg, dynamic baseline models, covariate adjustments). Improving accuracy will require multimodal devices capable of integrating photoplethysmography, electrodermal activity, and movement data.

Most nonmotor symptom monitoring studies remain at the pilot or experimental validation stage, with limited evidence from long-term deployment in routine clinical practice. Importantly, reported performance metrics vary considerably across studies due to differences in sensor types, monitoring duration, sample size, and validation settings; therefore, direct quantitative comparisons between studies are not appropriate. This heterogeneity constrains the generalizability of current findings and should be carefully considered when interpreting reported performance.

Overall, intelligent identification of nonmotor symptoms in PD remains an evolving field. Although existing studies demonstrate technical feasibility across multiple symptom domains, most systems have been validated in small-scale or short-term settings. Future research should prioritize longitudinal monitoring, multimodal integration, and clinically interpretable outputs to enhance translational value.

#### Clinical Decision-Making Limitations

Nonmotor monitoring demonstrates technical feasibility but limited clinical utility. Conceptually, sleep data could guide clinical decisions: rapid eye movement sleep behavior disorder detection might prompt melatonin or dopaminergic timing adjustments [35,37], sleep efficiency metrics could inform light exposure therapy [38], and reduced mobility might trigger fall prevention counseling [64]. Similarly, autonomic data could guide cardiovascular risk management [40] or orthostatic hypotension interventions [43].

However, these pathways remain largely theoretical. No study has demonstrated that acting on algorithm-detected sleep or autonomic abnormalities improves patient outcomes, reduces hospitalizations, or enables superior medication optimization compared with standard care. Key barriers include the inability to separate mixed symptom etiologies (eg, sleep fragmentation due to hypokinesia versus depression), the lack of validated thresholds linking specific values to specific actions, and the absence of prospective trials with hard clinical end points. At present, nonmotor monitoring primarily documents physiological patterns without supporting evidence for evidence-based therapeutic decisions.

These clinical limitations reflect deeper structural barriers. Nonmotor monitoring lags behind motor technologies in regulatory clearance and outcome trials [9,10,12]. Four structural barriers help explain this gap.

- Signal: motor symptoms produce direct measurements, whereas nonmotor proxies such as heart rate variability lack specificity.
- Validation: motor assessment relies on the reliable MDS-UPDRS part III, whereas nonmotor gold standards are often expensive or episodic [24].
- Environment: motor patterns are relatively stable, whereas nonmotor measures may degrade in home environments [24,33].
- Actionability: motor symptoms trigger clearer clinical decisions [9,12], whereas nonmotor data often have multiple causes without validated thresholds.

These fundamental barriers require new sensors, personalized baselines, and outcome trials—not just improved algorithms.

#### Interpreting the Metrics

The accuracy, AUC, and ICC values reported in this section represent technical validation results rather than evidence of clinical efficacy. Three limitations apply: studies used different methods and populations, preventing direct comparison; performance often declines in real-world use [35,41]; and no study has demonstrated that these measures improve patient outcomes. These figures illustrate technological potential but do not support conclusions about clinical readiness or comparative effectiveness.

### Multimodal Data Analysis Algorithms

The high-performance metrics reported for algorithmic approaches (eg, accuracy, sensitivity) must be interpreted within their methodological context. These results are typically achieved using curated datasets under constrained evaluation protocols, which may not fully represent the heterogeneity and noise encountered in continuous, real-world monitoring.

#### Single-Modality Feature Extraction

Single-modality approaches use one type of data or sensor to build models (Table S5 in Multimedia Appendix 3). Convolutional neural networks (CNNs), frequently combined with other algorithms, are prominent for extracting spatial features from movement data in PD [18]. San-Segundo et al [19] reported laboratory-based use of CNNs for tremor percentage estimation, achieving error rates below 5%. While this indicates strong performance under controlled conditions, laboratory-optimized algorithms often experience accuracy declines when deployed in uncontrolled home environments due to signal variability. Additionally, lightweight models tailored for wearable sensors are advancing. Borzì et al [20] combined simple thresholding with CNNs for home-based freezing-of-gait detection, achieving 96% sensitivity. However, sensitivity alone does not capture the full picture; specificity and real-time performance in varied daily contexts are equally critical for clinical utility.

Beyond classic motion sensors, modalities including eye tracking, plantar pressure, and voice analysis (eg, studies by Amato, Chudzik, and Tsakanikas [21,22,65]) are broadening the scope of single-modality PD feature extraction.

Despite their utility, single-modal techniques often yield lower accuracy in real-world, nonlaboratory conditions due to the variability of daily activities and the heterogeneity of PD symptoms. As demonstrated by Sigcha et al [18], consumer smartwatches and deep learning improve early-stage PD monitoring; however, additional validation is essential for advanced-stage disease with more pronounced symptoms. Error propagation remains a concern (eg, rest-period misclassification), requiring more parameters and increasing model complexity. Overall, single-modal approaches have limited predictive validity for the total UPDRS score, as they mainly quantify motor function [66]. For nonmotor symptoms such as sleep disturbances, precision remains to be optimized [23].

#### Cross-Modality Fusion Strategies

Multimodal intelligent monitoring integrates multiple heterogeneous data modes to achieve a comprehensive, continuous, and accurate assessment of PD. Multimodal integration relies on hierarchical data fusion, and common strategies mainly include feature-level fusion and decision-level fusion (see Table S6 in Multimedia Appendix 3).

In feature-level fusion, the research constructs a unified high-dimensional feature representation by integrating the original or intermediate features from different modes. For example, by simultaneously analyzing the patient’s voice signal and facial video features and using machine learning algorithms for modeling, the PD detection accuracy can reach as high as 83% [24]. In addition, to more comprehensively assess fall risk in a free-living environment, gait data and video eye-tracking data are combined. By using the fine-tuned YOLOv8 target detection algorithm to identify objects (such as obstacles and stairs) and potential hazards in the environment, and combining this with an overlap detection algorithm to analyze the intersection of the patient’s fixation point, walking path, and hazards, the approach can provide key environmental context information for gait fluctuations and enhance the perception of fall risk [25].

At the same time, techniques such as multigranularity computation, rough set theory, and composite algorithms strengthen robustness. For instance, one study reported 98.9% accuracy for gait activity recognition using a CNN-recurrent neural network ensemble [67]. Although this suggests excellent performance on their specific dataset, such high values often reflect optimization on curated, task-specific data and may not generalize to the heterogeneity of free-living conditions.

In decision-level fusion, models first classify or regress data from each modality independently and then integrate the outputs from individual models. For example, constructing a digital health technology composite score by combining sensor features from multiple active and passive tests can provide a more robust assessment of overall PD severity. In addition, machine learning models have been used to predict MDS-UPDRS total scores or distinguish “on/off” states by fusing multisource features, enabling multidimensional information integration and final decision-making. These methods demonstrate good reliability and validity in early-stage PD populations and provide a digital basis for tracking disease progression [27].

Cross-modal fusion faces many technical challenges, especially feature synchronization. Consistency across data streams is vital to suppress false classifications. Diverse sampling rates and structures necessitate complex temporal alignment. In addition, domain adaptation is indispensable to counteract device and environment biases, and data augmentation strategies help offset small sample limitations (eg, Shin et al’s [28] variable window shifting). These methods collectively help maintain stable performance across limited and heterogeneous datasets.

#### Federated Learning Applications

Federated learning—a distributed model training approach without raw data sharing—offers promise for cross-center collaboration in PD monitoring by enhancing privacy and scalability (see Table S7 in Multimedia Appendix 3). The study emphasizes that clinical data for PD encompass multimodal information on both motor and nonmotor features, such as images and wearable sensor data, providing abundant resources for federated learning applications [68]. The Diversity-Aware Activity Recognition framework, for example, clusters users by gait, activity, and social factors using K-means, enabling personalized model initialization and feature extraction [29]. This approach improves recognition of “cold-start” new users by 5.5%, and diversity-aware models demonstrate strong generalization across multiple activity tasks [29].

Key challenges include significant data distribution differences across users or devices, which affect the model’s generalization ability. The introduction of noise in differential privacy mechanisms may reduce data utility; therefore, it is necessary to further optimize the privacy budget and noise mechanisms to balance privacy and utility [29]. The deployment of federated learning frameworks on mobile devices is also limited by computing resources and energy consumption [29]. In addition, medical data are mostly unstructured and lack unified annotation, which affects the quality of model training, and the design and implementation of the entire system must comply with data privacy regulations such as the General Data Protection Regulation and HIPAA [68].

Future directions include the use of federated learning to promote multimodal intelligent monitoring of PD. First, promote multimodal feature fusion by integrating wearable sensors, voice, and image data [69]. Second, develop personalized and adaptive models and use meta-learning, transfer learning, and other technologies to help models better adapt to heterogeneous data [29]. Third, promote lightweight and edge-computing deployment of models to adapt to the resource constraints of mobile devices and enable real-time monitoring and feedback [29]. Fourth, carry out cross-center clinical verification and evaluate the effectiveness and safety of the federated learning system in real clinical scenarios through multiagency collaboration [68]. Continued advancements in multimodal symptom monitoring will be achieved by optimizing feature extraction and fusion, combined with deeper integration of federated learning for secure, efficient data sharing and model training. This convergence will drive systems toward greater intelligence, personalization, and clinical applicability, underpinning precision diagnosis and management of PD.

### From Technology to Translation: Challenges and Integrated Strategies for PD Remote Monitoring Platforms

#### Opportunities

The swift expansion of telemedicine has created ample opportunities for deploying intelligent monitoring technology in PD [43,70,71]. Since the advent of COVID-19, remote neurology consultations have increased by 17%. Notably, 98% of surveyed deep brain stimulation patients express a strong interest in remote management [46], representing a major catalyst for remote monitoring platforms. Accompanying these trends, acceptance rates for wearable and environmental sensors reach 87% and 100%, respectively [14], setting a robust social foundation for clinical translation.

The technical foundations have simultaneously matured: digital monitoring of PD symptoms demonstrates notable progress (PDMonitor tremor detection reports 99% concordance with MDS-UPDRS-III [10]), and cross-modal algorithmic optimization elevates fall risk prediction to 83% accuracy [13]. Together, these improvements unite hardware and software for reliable remote monitoring platforms.

#### Transformational Results

Remote monitoring platforms now span the entire continuum of PD management. Health care interaction–optimized platforms facilitate secure, efficient communication for individualized care (eg, MoveONParkinson achieves a System Usability Scale score of 79.50, 95% CI 73.70-85.30 [47]; RM App attains 85.5%, 172/201, adherence among deep brain stimulation patients [48]). Symptom-oriented platforms provide real-time physical symptom tracking and detailed assessment (QDG-Care and iHandU focus on tremor quantification [49,50], and PDxOne on gait analysis [51]). Comprehensive platforms, such as My Parkinsoncoach, integrate wearables and smartphones to collect data, deliver feedback, reduce outpatient visits by 29%, and enhance patient empowerment; solutions such as uMotif, Fox Wearable Companion, and PD Dr optimize medication tracking [72]. Nonetheless, integrated remote monitoring platforms encompassing both motor and nonmotor symptoms are still evolving—MoveONParkinson, for example, is limited to conversational interactions, while PDxOne focuses solely on gait. Such segmentation hinders holistic disease management and longitudinal care decisions.

Meanwhile, innovative rehabilitation solutions are broadening the implementation ecosystem for PD. Platforms such as “Shou Pa” incorporate Tai Chi to boost adherence, and initiatives such as ACTIVA, i-PROGNOSIS, and Rhythm Workers use virtual games for intensive motor training [53], illustrating a growing diversity of technological approaches.

#### The Care-Platform Transformation in PD (CPT-PD) Framework

While these platforms demonstrate technical excellence in their respective domains—including motor assessment, decision support, and treatment optimization—they are primarily focused on single functions or specific points in the care pathway. For example, SMaRT-PD provides a rule-based clinical decision support system that integrates multimodal data (wearable sensors, patient-reported outcomes, and care records) to generate personalized, symptom-specific management recommendations [54]. QDG-Care delivers validated quantitative metrics of motor symptoms through a fully integrated connected platform, with components spanning a dedicated digitography device, patient app, cloud-based algorithm service, provider dashboard, and electronic health record integration [49].

**Figure 2:**
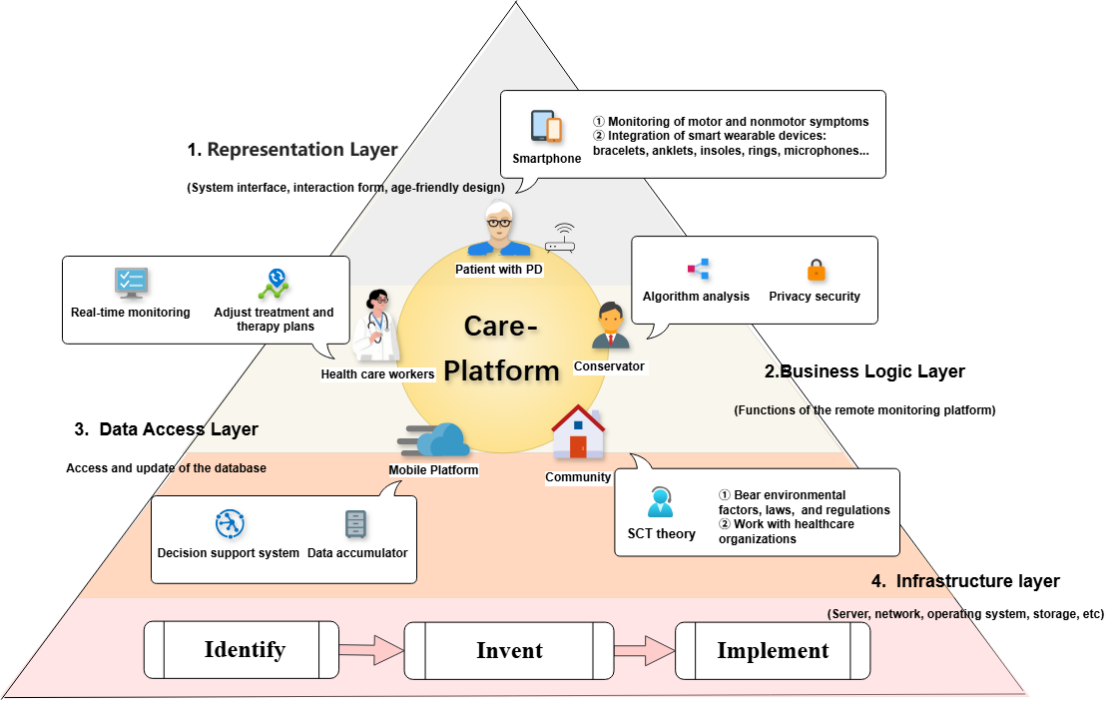
The Care-Platform Transformation in Parkinson Disease (CPT-PD) framework: a conceptual model integrating key components for the design of effective remote monitoring platforms. PD: Parkinson disease; SCT: social cognitive theory.

Identifying recurring challenges and themes in existing platforms informs the development of the Care-Platform Transformation in PD (CPT-PD) framework, which synthesizes key ingredients for effective PD monitoring (Figure 2). This is a reference model that draws on retrospective lessons rather than offering prescriptive advice and is intended to support the design of holistic, integrated remote monitoring platforms (Textbox 1).

**Framework for the reference model.**

**1. Longitudinal, clinically driven pathway—identify, invent, implement**

Initially, stakeholder needs—including monitoring efficiency, symptom assessment, and doctor-patient interaction—are clarified [46]. Clinical pilot validation and iterative algorithm development follow, culminating in the evaluation of health outcomes and cost-effectiveness for platform deployment.

**2. Four-layer horizontal architecture**

User-centric design in the presentation layer prioritizes accessibility (eg, large font, intuitive controls) for older patients with PD. The business logic layer emphasizes core symptom monitoring and facilitates clinician-patient exchanges. The data access layer upholds regulatory compliance and multimodal data security, leveraging frameworks such as Fast Healthcare Interoperability Resources and encrypted transmission [55]. The infrastructure layer provides essential network support for system reliability.

**3. Collaborative Five-Element Model**

Patients with PD submit multimodal data; health care workers use these data for decision-making; administrators ensure platform operation; integrated remote monitoring platforms support system functions; and the community provides supportive networks. Together, these elements interlink into a closed-loop “diagnosis-treatment-management” intelligent management cycle.

The synthesized framework (Textbox 1) offers a structured tool for analyzing platform strengths and weaknesses and a road map for overcoming fragmentation and enhancing clinical integration.

To validate the clinical applicability of this framework, we present the “My Parkinsoncoach” platform, which has successfully reduced outpatient burden in PD. This case example demonstrates how it organically integrates the longitudinal pathway, horizontal architecture, and collaborative ecosystem of the CPT-PD framework [52].

The platform’s development clearly follows the longitudinal, clinically driven pathway: it began by identifying the unsustainable burden of frequent outpatient visits; invented an integrated solution comprising periodic electronic questionnaires, visual symptom-domain scoring, clinician-patient communication, and educational modules; and implemented the system through a prospective cohort study that verified a 29% reduction in outpatient visits and a 39% decrease in health care costs. This structured development process is a key reason for the platform’s success.

We now explain the platform through the horizontal 4-layer architecture: its presentation layer provides role-specific, user-friendly interfaces for patients and clinicians; the business logic layer automatically converts questionnaire responses into trend scores across 15 symptom domains (eg, motor symptoms, activities of daily living) and implements a clinical response protocol whereby specialized nurses handle initial alerts and neurologists provide backup; the data access layer securely manages these multimodal patient-reported data; and the entire system operates on a stable infrastructure layer (network connectivity).

Within this architecture, the 5 key collaborative elements work in synergy to sustain stable platform operation: patients submit data via the mobile interface; specialized nurses and neurologists interpret the data and intervene based on prompts from the business logic layer; platform operators ensure system stability; the platform itself serves as the integrated functional carrier; and the broader community (eg, family caregivers)—although not highlighted in the original study—represents a natural extension for scaling the model into home-based care.

Applying this case to the CPT-PD framework demonstrates its ability to deconstruct the design logic and core components of a successful platform. At the same time, it reveals areas for framework refinement—notably, the integration of the “community” element remains underdeveloped. This insight points the way toward future optimization of both the “My Parkinsoncoach” platform and the CPT-PD framework itself.

#### Transformational Bottleneck

Despite progress, only a minority of monitoring devices have transitioned into remote monitoring platforms. This translational bottleneck fundamentally stems from a mismatch between reported performance and clinical readiness. While many studies demonstrate promising results in controlled pilot settings (Technology Readiness Level ~4-5), only a few progress to robust validation in heterogeneous, real-world clinical environments (Technology Readiness Level 7-8), creating a “readiness gap” that hinders broad adoption [73]. This gap is compounded by several persistent bottlenecks:

- Clinical research needs greater incorporation of patient- and community-centered social cognitive theory [47,56], increasing primary care collaboration to prioritize key patient needs.
- Technical fragmentation results in isolated symptom focus and insufficient holistic assessment, and the detachment of development from clinical practice undermines nonmotor symptom monitoring [74].
- Device and algorithm limitations stem from a lack of standardization—the PKG and STAT-ON offer inconsistent data structures, forcing manual curation by clinicians and increasing errors. Artificial intelligence (AI)–sensor integration faces data standardization and regulatory obstacles, as well as extensive validation requirements [75], requiring collaboration among neurologists, AI experts, and regulators.
- Low smartphone adoption among older adult patients necessitates cognitive load–sensitive, age-friendly design with voice user interfaces, simple user interfaces [57], and home assistive device compatibility [71,76-78]. This challenge is particularly evident in studies such as eSanjeevani, which reported low adherence to wearable devices among older patients with PD in India, with health care providers citing “low penetration rates and insufficient convenience (especially among the elderly)” [78]. Notably, only 32.4% of compiled smart device studies used randomized controlled trials focusing on clinical experience, highlighting a research-to-practice gap [79]. Addressing these issues demands interdisciplinary breakthroughs in algorithms and a paradigm shift toward patient-centered, senior-accessible design.

## Discussion

This review provides a critical evaluation of the latest advancements and persisting challenges in multimodal intelligent monitoring for PD. However, as highlighted in our terminology (Table 1), the field currently includes many multisensor systems with limited algorithmic fusion. Around the 3 core dimensions proposed in the “Introduction” section, the main findings of this study are as follows. First, in terms of the clinical performance of wearable sensors, the multisite deployment of IMUs and new sensing technologies has significantly improved the objectivity and continuity of motor symptom monitoring, but wearing comfort, long-term compliance, and data standardization remain the main obstacles to clinical translation. Second, at the level of algorithms and methods, single-mode feature extraction technologies perform well under laboratory conditions, but their accuracy declines in real-world environments. Cross-modal fusion strategies enable more comprehensive disease phenotype analysis by integrating multisource data. Distributed computing frameworks such as federated learning show potential for multicenter collaboration under the premise of ensuring data privacy. Third, in terms of the clinical translation of remote monitoring platforms, existing platforms cover multiple dimensions such as medical interaction optimization, symptom-oriented monitoring, and comprehensive management, but integrated platforms that truly achieve coordinated monitoring of motor and nonmotor symptoms are still in the early stages of development.

Unlike prior reviews focusing on isolated technologies, we adopt an integrative perspective encompassing the quantification of both motor and nonmotor symptoms, the development of analytical algorithms, and the evolution toward remote monitoring platforms. Evidence indicates that platforms integrating wearable sensors, computer vision, and biosignal analytics can substantially enhance objectivity and continuity in symptom tracking (see Tables S2-S5 in Multimedia Appendix 3). Advanced multimodal algorithms and distributed computing demonstrate potential strengths in secure, multicenter data sharing, privacy protection, and model generalizability (see Tables S6 and S7 in Multimedia Appendix 3), in addition to improving medication adherence [72]. The widespread adoption of remote monitoring platforms further validates the clinical value of a closed-loop “diagnosis-treatment-management” approach, optimizing health care resource use and reducing outpatient burden [52,72].

The heterogeneity documented across studies—in technologies, populations, protocols, and outcome measures—while reflecting a vibrant and innovative field, presents a primary challenge for synthesizing evidence and translating devices into practice. This variability precludes simple quantitative comparisons and complicates the assessment of a technology’s true clinical readiness. To move beyond this stage, future research should prioritize standardization (eg, validation protocols against common clinical benchmarks), harmonization (of data formats and outcome measures), and pragmatic study designs that evaluate performance in intended-use settings with representative patient populations. Addressing these methodological challenges is as crucial as algorithmic innovation for realizing the potential of multimodal monitoring.

Despite these promising developments, several persistent obstacles impede translation into practice. A major limitation is that most existing solutions remain focused on narrow symptom domains, lacking coordinated quantification across motor and nonmotor features [33]. Moreover, algorithmic adaptability is constrained by heterogeneity in devices and data, underscoring the need for robust multicenter validation [68]. Finally, practical barriers to widespread adoption include platform complexity and low rates of smart device usage among older adult populations [58].

The heterogeneity recorded across studies in technology, population, protocols, and outcome measures—although reflecting the vigorous innovation in this field—also brings systemic challenges for evidence integration and clinical translation. To move beyond this stage, the field must address several key research gaps. Based on the comprehensive evidence in this review, we identified 5 key gaps that, if addressed, would accelerate progress:

First, and most fundamental, there is a lack of longitudinal real-world validation. The vast majority of studies are still limited to short-term, small-sample, laboratory or semicontrolled verification, which fails to capture the progressive nature of PD and lacks longitudinal follow-up data for months or even years in real home environments. As Espay et al [58] stated, this leads to an extremely limited understanding of the long-term compliance with technology, data stability, and its dynamic association with disease progression. Therefore, conducting longitudinal real-world validation has substantial clinical value and cost-effectiveness.

Second, at the system level, current intelligent monitoring generally remains fragmented, focusing on individual symptoms. The lack of integrated platforms quantifying interactions among motor fluctuations, sleep, mood, and autonomic function creates a bottleneck, limiting progression from symptom monitoring to holistic disease management. In view of the high variability of PD symptoms, it has been proposed to develop “multidomain, multisensor, intelligent technologies” to “identify individual disease fingerprints and achieve truly personalized treatment” [58].

Third, from sensor deployment to algorithm performance and clinical reference standards, studies lack unified normative frameworks and exhibit high methodological heterogeneity. The absence of standardized clinical outcome indicators makes it extremely difficult to compare the performance of different technologies or synthesize data across studies and also hinders regulatory approval of digital outcome indicators by agencies such as the Food and Drug Administration [33,58,73,75].

Fourth, although patient-centered design has been repeatedly emphasized, existing intelligent monitoring platforms still show deficiencies in user interface friendliness, cognitive load management, and aging adaptation when serving the older adult population with a high incidence of PD. This has directly led to low technology acceptance and poor long-term compliance in the target group [57,58,78].

At a deeper level, existing research has focused too much on improving prediction accuracy through feature- or decision-level fusion but has largely ignored the pathophysiological logic underlying multimodal data [58]. In fact, true multimodal intelligence should be able to explain the interactions among different modalities rather than simply pursuing higher classification accuracy.

Therefore, addressing these methodological and translational gaps is as critical as algorithmic innovation to realize the potential of multimodal monitoring. To overcome these challenges, future research should prioritize the development of an integrated, precise, and scalable intelligent management ecosystem for continuous PD monitoring. A phased approach is proposed to guide this effort.

In the short term, emphasis should be placed on the coevolution of hardware and algorithms. On the one hand, efforts are needed to develop more wearable, comfortable, and multimodal sensing hardware capable of long-term continuous monitoring, integrating diverse data streams such as sleep patterns, heart rate variability, and speech [13,30,35,40,42,45,56]. On the other hand, at the algorithmic level, capabilities in signal processing and multimodal information fusion must be strengthened, leveraging adaptive fusion mechanisms to enable deeper disease phenotyping [29,64,66,67,80]. Over the medium to long term, the establishment of a secure and compliant multicenter collaborative framework is essential. This will require the implementation of privacy-preserving computational techniques, such as federated learning, to aggregate knowledge from localized data processing securely, alongside embedded privacy-enhancing technologies to meet regulatory and ethical requirements [68,81]. Concurrently, in advancing toward international standards, it is critical to institute dynamic patient-informed consent mechanisms and proactive governance—not merely to fulfill legal obligations but to cultivate a trustworthy, patient-centered, and privacy-aware intelligent platform [68,81].

Importantly, insights drawn from the CPT-PD framework highlight that system design and deployment must thoroughly account for the needs of older users. Consequently, future platforms should integrate AI-driven clinical decision support with aging-adaptive design principles, employing user-friendly devices, stepwise low-burden interactions, and clear, timely feedback to minimize cognitive and operational demands. Such an approach is key to enhancing compliance and achieving truly patient-centric, progressive health management [78,82].

In summary, the innovation of this study lies in its departure from merely commenting on a single technology or symptom dimension. Instead, based on existing evidence, it pioneers a unified framework, CPT-PD, that integrates multimodal perception, edge intelligence, privacy computation, and aging-adaptive design. More importantly, by clearly identifying key research gaps in longitudinal effectiveness, symptom fragmentation, and lack of standardization, this review provides a prioritized road map. In stark contrast to existing research that typically focuses on the performance of specific algorithms or the validation of single devices, this study emphasizes the collaborative quantification of motor and nonmotor symptoms, the coevolution of hardware and algorithms, and the deep integration of technology and humanistic care. Consequently, the key contribution of this study to the field is that it provides a clear road map and theoretical foundation for PD management to transition from a fragmented, technology-centric “tool accumulation” phase to a systematic, patient-centered “intelligent ecosystem.” In practical terms, this means we are poised to overcome the bottlenecks of low technology conversion rates and poor patient acceptance. By constructing a truly accessible, reliable, and user-friendly monitoring system for older adults, we can shift from passive diagnosis and treatment to proactive, continuous home health management. This, in turn, will tangibly improve patients’ quality of life, reduce the overall health care burden, and ultimately advance the goals of precision medicine and scalable, patient-centered disease management.

## Data Availability

All data generated or analyzed during this study are included in this published article or its multimedia appendices.

## Appendix

Multimedia Appendix 1 Detailed search strategy and process.

Multimedia Appendix 2 Detailed search strategy and process.

Multimedia Appendix 3 PRISMA-ScR (Preferred Reporting Items for Systematic Reviews and Meta-Analyses extension for Scoping Reviews) checklist.

## Abbreviations

AI: artificial intelligence
AUC: area under the curve
CNKI: China National Knowledge Infrastructure
CNN: convolutional neural network
CPT-PD: Care-Platform Transformation in PD
GAI: generative artificial intelligence
GAIDeT: Generative Artificial Intelligence Delegation Taxonomy
HIPAA: Health Insurance Portability and Accountability Act
ICC: intraclass correlation coefficient
IMU: inertial measurement unit
MDS: Movement Disorder Society
mHealth: mobile health
PD: Parkinson disease
PKG: Personal KinetiGraph
PRISMA: Preferred Reporting Items for Systematic Reviews and Meta-Analyses
PRISMA-S: Preferred Reporting Items for Systematic Reviews and Meta-Analyses Literature Search Extension
PRISMA-ScR: Preferred Reporting Items for Systematic Reviews and Meta-Analyses Extension for Scoping Reviews
UPDRS: Unified Parkinson Disease Rating Scale
