# Supplementary material for: Multimodal Intelligent Monitoring of Parkinson Disease: Scoping Review of Progress and Translational Challenges
Source: J Med Internet Res. 2026 Apr 17;28:e89057. doi: 10.2196/89057 (PMC13135170; doi:10.2196/89057)
Supplement: Multimedia Appendix 1 [file jmir_v28i1e89057_app1.docx]

**Multimedia Appendix 1**

Detailed Search Strategy and Process

1. Overview and Methodology

The literature search for this scoping review was designed and reported following two key methodological frameworks: the three-step iterative search strategy recommended by the Joanna Briggs Institute (JBI) for scoping reviews to maximize comprehensiveness; and the PRISMA-S (Preferred Reporting Items for Systematic reviews and Meta-Analyses literature search extension) guidelines to ensure transparent and reproducible reporting of all search components.

2. JBI Three-Step Search Strategy: Detailed Process

Step 1: Initial Limited Search & Analysis

An initial limited search was performed in PubMed (via NCBI) and Web of Science (via Clarivate) in August 2025. The aim was to identify **some** relevant keywords, index terms (e.g., Medical Subject Headings [MeSH] in PubMed), and the breadth of terminology used in the field. Analysis of the titles, abstracts, and indexing of retrieved articles informed the refinement of the final search vocabulary.

Key concepts identified and subsequently used in the formal search included: Parkinson’s disease, wearable sensors, artificial intelligence, remote monitoring, telemedicine, and multimodal.

Step 2: Formal Systematic Search

Based on the refined vocabulary from Step 1, a formal systematic search was executed across all selected databases. The search strategy was tailored to the syntax and features of each database (e.g., MeSH in PubMed, Topic fields in Web of Science). Searches covered the period from January 1, 2019, to December 31, 2024. An update search was run on January 22, 2026, to capture any records published online ahead of print between the final search date and the analysis phase. No language filters were applied at this stage. The complete search strings used in each database are provided in Table 1 below.

Step 3: Supplementary Searching

To minimize the risk of omitting relevant studies, one supplementary search methods were employed--- Citation Snowballing: The reference lists of all 66 included studies and 13 key reviews identified during screening were manually examined for additional relevant sources.

3. Search Results and Complete Search Strings

The table below details the databases, platforms, dates of the formal search (Step 2), the exact search strings used, and the number of records initially retrieved. These numbers correspond to the “Identification” phase in the PRISMA-ScR flow diagram (Figure 1 in the main manuscript).

Table 1. Details of each database search strategy

| Database & Platform | Date Searched | Search Parameters (with concept groups and field tags) | Initial Number |
| --- | --- | --- | --- |
| PubMed (via NCBI) | 2025-08-15 (Updated: 2026-01-22) | #1 Population: ("Parkinson Disease"[Mesh] OR "Parkinson's disease"[TIAB] OR "Parkinson disease"[TIAB] OR PD[TIAB])  #2 Sensing modalities: ("Wearable Electronic Devices"[Mesh] OR "wearable sensor"[TIAB] OR "wearable device"[TIAB] OR "inertial measurement unit"[TIAB] OR IMU[TIAB] OR "computer vision"[TIAB] OR "speech"[TIAB])  #3 Intelligent methods: ("Artificial Intelligence"[Mesh] OR "machine learning"[TIAB] OR "deep learning"[TIAB] OR "algorithm"[TIAB] OR "multimodal"[TIAB] OR "data fusion"[TIAB] OR "digital biomarker"[TIAB])  #4 Platform level: ("Telemedicine"[Mesh] OR "remote monitoring"[TIAB] OR "telemedic"[TIAB] OR "mHealth"[TIAB] OR "digital health"[TIAB])  Final: #1 AND (#2 OR #3 OR #4) AND (2019/01/01:2024/12/31[pdat]) | 1175 |
| Web of Science(via Clarivate) |  | 1 Population: TS=("Parkinson* disease" OR PD)  2 Sensing modalities: TS=("wearable sensor" OR "wearable device" OR "inertial measurement unit*" OR IMU OR "computer vision" OR speech)  3 Intelligent methods: TS=("artificial intelligence" OR "machine learning" OR "deep learning" OR algorithm* OR multimodal OR "data fusion" OR "digital biomarker*")  4 Platform level: TS=("remote monitoring" OR telemedic* OR mHealth OR "digital health")  Final: #1 AND (#2 OR #3 OR #4)  Limits: PY=(2019-2024) AND DT=(Article OR Review) | 2256 |
| CNKI |  | 1 Population: (主题：帕金森病 OR 主题：帕金森)  2 Sensing modalities: (主题：可穿戴 OR 主题：传感器 OR 主题：计算机视觉 OR 主题：语音)  3 Intelligent methods: (主题：人工智能 OR 主题：机器学习 OR 主题：深度学习 OR 主题：算法 OR 主题：多模态 OR 主题：数据融合 OR 主题：数字生物标志物)  4 Platform level: (主题：远程监测 OR 主题：远程医疗 OR 主题：移动健康 OR 主题：数字健康)  Final: #1 AND (#2 OR #3 OR #4) NOT (主题：MRI OR 主题：生物标记物)  Limits: 发表时间：2019-01-01至2024-12-31; 文献类型：期刊论文、综述 | 152 |

* The search terms were organized into four conceptual blocks to ensure comprehensive coverage of the multidisciplinary field: disease population, sensing hardware, analytical methods, and system-level platforms. Within each block, synonyms and variant spellings were included based on terminology identified in key papers and database thesauri. The Boolean operator OR was used within blocks to capture all relevant terms, and AND was used between blocks to ensure studies addressed at least one aspect from each conceptual area relevant to multimodal intelligent monitoring.
