# Supplementary material for: Multimodal Intelligent Monitoring of Parkinson Disease: Scoping Review of Progress and Translational Challenges
Source: J Med Internet Res. 2026 Apr 17;28:e89057. doi: 10.2196/89057 (PMC13135170; doi:10.2196/89057)
Supplement: Multimedia Appendix 2 [file jmir_v28i1e89057_app2.docx]

**Multimedia Appendix 2**

Table 1. Technical parameters of typical wearable sensor devices

| Device Name | Core Technology | Wearing Location | Evaluation Indicators | Key Performance Verification Results |
| --- | --- | --- | --- | --- |
| PDMonitor® | 9-degree Inertial Measurement Unit (IMU) Sensor (Accelerometer, Gyroscope, and Magnetometer) | Wrist (bilateral), Ankle (bilateral), Waist | Movement disorders, bradykinesia, gait, tremor, dyskinesia, on-off fluctuations, etc. | Bradykinesia: accuracy 0.85, specificity 0.85, sensitivity 0.84 (UPDRS items 23+24+25 > 4)  Gait Dysfunction: Accuracy 0.99, Specificity 1.00, Sensitivity 0.67 (UPDRS Item 29 > 1)  Tremor (wrist): accuracy 0.99, specificity 0.99, sensitivity 0.84; Tremor (leg): accuracy 0.99, specificity 0.99, sensitivity 0.93 (UPDRS item 20 > 1)  Dyskinesia: accuracy 0.99, specificity 0.99, sensitivity 0.82 (AIMS > 4)  OFF state detection: accuracy 0.96, specificity 0.97, sensitivity 0.85 (based on diary and expert assessment, correlation r²=0.75) |
| PKG® Watch | 3-axis IMEMS accelerometer (ADXL345 Analog Devices) | Wrist | Movement Fluctuations, Dyskinesia, Bradykinesia, etc. | Bradykinesia: accounts for 50% of symptoms identified by PKG but not reported by patients; accounts for 61% of all treatable findings  Dyskinesia: 33% of symptoms identified by PKG but not reported by patients; 32% of all treatable findings  Tremor: 17% of symptoms identified by PKG but not reported by patients  Symptom identification consistency: In 35% of visits, PKG reported symptoms that patients did not actively report; 17.6% of patients reported symptoms that were not reflected in the PKG  Impact of treatment adjustments: In 84% of visits (79% of patients), PKG data led to 74 treatment plan changes |
| STAT-ON™ | 9-degree Inertial Measurement Unit (IMU) Sensor (Accelerometer, Gyroscope, and Magnetometer) | Waist | Movement Fluctuations, Dyskinesia, Freezing of Gait, etc. (Tremor is not recorded) | Movement Fluctuations: Detection Rate in Suspected Patients: 95.4%  Freezing of Gait: High Consistency with Patient Self-Reports (16 patients suspected of having Freezing of Gait were detected, of which 14 were confirmed to have self-reported conditions)  Predicted treatment decision: Highly consistent with final SLT treatment decision (φ coefficient = 0.73, *p* < 0.001) |
| Kinesia 360™ | Wireless Motion Sensor | Wrist, Ankle | Tremor, Dyskinesia | UPDRS III Improvement: -5.3 points in the EG group, -1.0 points in the CG group (p=0.134)  UPDRS II improvement: experimental group (EG) -2.1 points vs control group (CG) +0.5 points (p=0.004)  The EG group had significantly more rotigotine dose adjustments than the CG group: 2.8 vs 1.8 |
| FeetMe® Monitor | Pressure Sensor, Insole IMU (3-D Accelerometer and 3-D Gyroscope) | Insole | Plantar pressure, gait parameters (speed, stride length, cadence, etc.), fall risk | Speed, Step Length, Cadence: Intraclass Correlation Coefficient (ICC) > 0.95 in all test conditions (single step, 8 m, 24 m)  Stance phase duration: paretic side vs. non-paretic side: ICC: 0.77-0.94  Swing phase duration: paretic side: ICC: 0.75 - 0.90 ; non-paretic side: ICC: 0.32 - 0.57 |

Table 2. Typical System Parameters of Computer Vision Technology

| System Name | Camera Type | Core Technology Input | Action Type | Evaluation Metric Validation Results |
| --- | --- | --- | --- | --- |
| Video Analysis System Based on MediaPipe | Intel RealSense D455 | MediaPipe 2D Pose Estimation Framework + Custom Motion Sequence Algorithm | MDS-UPDRS 3.4–3.8 five limb bradykinesia movements | Evaluation metrics: average period, average amplitude, total duration, period slope, amplitude slope, period SNR, amplitude SNR  Motion parameters are significantly correlated with MDS-UPDRS:  Finger tapping: period SNR (ρ=0.361, p<0.01) and amplitude SNR (ρ=0.361, p<0.01);  Hand movement: amplitude SNR (ρ=0.303, p<0.05);  Forearm rotation: mean amplitude (ρ=-0.429, p<0.01);  Sensitivity: can distinguish PD patients with MDS-UPDRS score of 0 from healthy subjects |
| KELVIN™ (based on OpenPose) | Smartphone, Tablet | OpenPose 2D pose estimation + random forest classifier | MDS-UPDRS 3.4–3.8 five limb bradykinesia movements | Evaluation Metrics: Speed, Amplitude, Hesitation/Pause, Signal Attenuation  Highly consistent with clinical scores: intraclass correlation coefficient ICC = 0.74 (p < 0.001);  84% of evaluation differences were less than clinically important;  Validated in 628 patients across 5 centers. |
| MIT radio respiratory monitoring system | Respiratory Belt + Radio Sensor, Non-Contact, No-Camera | Core Technology: RF Signal Processing + Custom Neural Network (Including Attention Mechanism and qEEG Auxiliary Tasks)  Input: Nighttime Respiratory Signal | Respiratory Signal | PD Diagnostic Performance:  Wireless Data: AUC = 0.906 (Sensitivity 86.23%, Specificity 82.83%);  Respiratory Belt Data: AUC = 0.889 (Sensitivity 80.22%, Specificity 78.62%);  External Validation (Mayo Clinic Data): AUC = 0.851.  PD Severity Assessment:  The model-predicted MDS-UPDRS total score is highly correlated with the clinical score: R = 0.94 (P = 3.6×10⁻²⁵)  Correlation with Each MDS-UPDRS Item:  Part I (Non-motor symptoms): R = 0.84  Part II (Motor Symptom Experience): R = 0.91  Part III (Motor Examination): R = 0.93  Part IV (Motor complications): R = 0.52 (because most patients have this sub-item as 0)  Early Risk Prediction: 75% of preclinical patients were identified early;  ICC > 0.95 after integrating data from multiple nights. |

Table 3. Comparison table of sleep disorder technologies

| Literature | Monitoring objectives | Device type | Measurement parameter function | Key performance | Study design | Participants | Monitoring setting | Reference standard | Validation level |
| --- | --- | --- | --- | --- | --- | --- | --- | --- | --- |
| Oz, S., et al. | Sleep stages | Soft electrode array | EEG EOG EMG signals | High consistency with vPSG (κ=0.688), REM sleep sensitivity 76.8% | Laboratory-based validation study comparing wearable EEG with vPSG | Healthy adults (n = 20) | Overnight laboratory-based monitoring | vPSG | Algorithm validation against gold standard |
| Raschellà, F., et al. | REM Sleep Behavior Disorder (RBD) | Wrist actigraph (GENEActiv Original) | Nighttime movement pattern analysis | Clinical test accuracy reached 92.9±8.16%; in home records, the accuracy was 100% in the 2-week window for PD patients and 94.4% for non-PD controls. | Diagnostic validation study | PD patients (n=30) and non-PD controls (n=20) | Home-based monitoring for 14 days | PSG-confirmed clinical diagnosis of RBD | Home-based clinical screening validation |
| Madrid-Navarro, C.J., et al. | Sleep quality and circadian rhythm | Home Cycle Monitoring (ACM) Device Kronowise 3.0 | Wrist temperature, triaxial acceleration, visible light exposure | There was no significant difference between the ACM device and PSG in monitoring parameters such as sleep efficiency. | Pilot validation study | Patients with Parkinson’s disease (n = 15) | Home-based ambulatory monitoring under free-living conditions | Polysomnography | Feasibility and agreement validation against gold-standard PSG |
| Obayashi, K., et al. | Light and sleep quality | Wrist Light Sensor (Actiwatch 2) + Bedroom Sensor (TR-74Ui and LX-28SD) | Light intensity, exposure time + activity recording | Insufficient daytime light and excessive nighttime light both reduce sleep efficiency | Observational cross-sectional study (PHASE study) | PD patients (n = 31) | Home-based monitoring under free-living conditions | Actigraphy-derived objective sleep parameters | Exploratory association analysis without PSG validation |
| Mirelman, A., et al. | Frequency of tossing and turning at night | Waist Accelerometer (Axivity AX3 or DynaPort MiniMod Module Device) | Data on nocturnal turning and axial movements in bed | Reduced tossing and turning is associated with worsening motor symptoms and autonomic nervous system disorders | Observational comparative study | PD patients (n = 33) and healthy controls (n = 22) | Home-based nocturnal monitoring under free-living conditions | Video-polysomnography (vPSG) | Nocturnal movement characterization using gold-standard vPSG |
| Wu, J.Q., et al. | Correlation between sleep and daytime function | Activity bracelet (ActiGraph GT9X Link) + smartphone | Actigraphy + Sleep Diary Questionnaire | Revealing a bidirectional association between subjective sleep quality and next-day anxiety | Observational longitudinal EMA study | PD patients (n = 29) | Smartphone-based real-world monitoring in daily life | Self-reported sleep and daytime functioning measures | Exploratory temporal association analysis |
| Gnarra, O., et al. | Sleep position and movement disorders | Sleep Profiler Headband | EEG EOG EMG + Head Position | Supine position duration is positively correlated with the severity of movement disorders | Exploratory observational study | PD patients (n = 20) | Home-based overnight monitoring under free-living conditions | Wearable-derived sleep architecture and head position metrics | Exploratory association analysis without gold-standard validation |

| Literature | Monitoring objectives | Device type | Measurement parameter function | Key performance | Study design | Participants | Monitoring setting | Reference standard | Validation level |
| --- | --- | --- | --- | --- | --- | --- | --- | --- | --- |
| Carrón, J., et al. | Voice analysis provides support for diagnosis | Server-Client Architecture, Android Application | Continuous sound (a vowel) | Accuracy in a controlled environment is 92%, while it drops to 71% in an uncontrolled environment | Methodological feasibility and usability evaluation study | Patients with Parkinson’s disease (n = 20) and healthy controls (n = 20) | Smartphone-based voice recording under controlled laboratory and uncontrolled real-world conditions | Diagnosis of Parkinson’s disease as reported by clinicians | Exploratory feasibility and usability assessment without formal clinical-scale validation |
| Suzuki, M., et al. | HRV | Polar V800 watch + H10 chest strap | 24-hour heart rate monitoring | AUC for Minimum HRV Value for Diagnosing PD = 0.90 | Cross-sectional observational study | Patients with Parkinson’s disease (n = 28) and healthy controls (n = 20) | Wearable sensor–based heart rate monitoring conducted under resting conditions in a controlled experimental setting | Clinical diagnosis of Parkinson’s disease with group-level comparison against healthy controls | Physiological signal–level validation based on group comparisons, without validation against standardized autonomic or non-motor symptom scales |
| Polverino, P., et al. | OH | Portable multi-parameter monitor | Blood Pressure, Blood Oxygen, and Body Temperature + Smart Alarms | Remote identification of OH episodes (high availability), high patient satisfaction | Prospective pilot telemonitoring study | Patients with Parkinson’s disease and orthostatic hypotension (pilot cohort; sample size not explicitly reported) | Real-time home-based telemonitoring using wearable vital-sign sensors and an ICT platform during daily living conditions | Clinical diagnosis of Parkinson’s disease and orthostatic hypotension based on routine blood pressure assessment criteria | Home-based physiological and symptom-level evaluation with real-time clinical oversight, without validation against standardized autonomic or non-motor symptom scales |
| Almeida, J.S., et al. | Sound Detection of PD | Acoustic Cardioid Microphone and Smartphone | Continuous Voice Signal | Professional Microphone Accuracy: 94.55%, Smartphone Accuracy: 92.94% | Retrospective machine learning–based diagnostic study using an existing speech dataset | Parkinson’s disease patients (n = 64) and healthy controls (n = 35) | Controlled recording setting using sustained phonation and speech tasks | Clinical diagnosis of Parkinson’s disease with disease stage 1–2.5 according to the Hoehn and Yahr scale | Algorithmic performance evaluation based on hold-out validation using accuracy, AUC, and equal error rate, without external clinical validation |
| Lim, W.S., et al. | Early PD Screening | Smartphone Camera (SJCAM SJ4000) + Microphone | Voice + Facial Expression Analysis | Comprehensive AUC = 0.85 for distinguishing early PD patients from controls; optimal diagnostic value +0.90 | Cross-sectional machine learning–based diagnostic study with independent training and validation cohorts | Patients with Parkinson’s disease (n = 186) and healthy controls (n = 185) | Smartphone-based recording of voice and facial expressions during a structured reading task | Clinical diagnosis of Parkinson’s disease according to the UK Parkinson’s Disease Society Brain Bank diagnostic criteria, with disease severity assessed using the Hoehn and Yahr scale | Algorithmic diagnostic validation using internal training–validation datasets with AUROC-based performance evaluation, without prospective or longitudinal clinical validation |
| Kyritsis, K., et al. | Dysphagia Assessment | Huawei Watch 2 | Hand Movement Delay for Tableware Handling | Clinical AUC=0.748, Real-World AUC reaches 1.000 | Observational sensor-based validation study conducted in both controlled clinical and free-living settings | Parkinson’s disease patients and healthy controls across three datasets: clinical dataset (21 PD, 7 HC), in-the-wild dataset EaH (10 PD, 4 HC), and long-term in-the-wild dataset SaH (3 PD, 3 HC) | Wearable smartwatch–based monitoring during meal consumption in clinical and free-living environments | Clinical diagnosis of Parkinson’s disease provided by movement disorders specialists, supported by UPDRS assessment | Validation of a sensor-derived eating behavior indicator through comparison with video ground truth and classification performance evaluation in clinical and real-life datasets |
| Wamelen, D.J., et al. | Correlation Between Motor and Non-Motor Symptoms | PKG Recorder | Wrist Acceleration | Quantitative Correlation Between Bradykinesia and Constipation | Retrospective exploratory observational study | 108 patients with idiopathic Parkinson’s disease (72 male, 36 female | Home-based continuous monitoring for 6 consecutive days | Clinical non-motor symptom scales (NMSS, PDSS, HADS, ESS, MMSE) | Exploratory association analysis between wearable-derived motor metrics and non-motor symptom domains |
| Schalkamp, A.-K., et al. | Prediction of Non-Motor Symptoms | Verily Smartwatch | Active Sleep Vital Signs | Digital Indicators Correlate with Autonomic Nervous Function, Limited Individual Prediction | Observational longitudinal cohort study | Patients with Parkinson’s disease from the PPMI cohort (n = 149 PD participants; 85 with overlapping clinical and digital data) | Free-living, at-home monitoring | Clinical non-motor and motor assessments collected during in-clinic visits, including cognitive, autonomic, psychiatric, daily living, and UPDRS-based measures | Association and longitudinal correlation with clinical reference measures (no diagnostic classification or individual-level prediction) |
| Lavorgna, L., et al. | Digital Phenotype Framework | Commercial Device (E4 Fitbit Oura) | Heart Rate and Activity Electrodermal Signals | Combining Traditional Assessment to Support Clinical Decision-Making | Mixed-method, cyclic, prospective observational study protocol | Older adults and patients with Parkinson’s disease planned for enrollment in the ActiveAgeing study (target sample size: approximately 200 participants) | Long-term free-living monitoring using wearable devices and smartphone-based assessments | Clinical assessments, patient-reported outcomes, and standardized geriatric and neurological scales planned as reference measures | Study protocol describing planned digital phenotyping and artificial intelligence analyses; no validation results reported |

Table 4. Comparison Table of Autonomic Dysfunction Technologies

Table 5. Comparison Table of Unimodal Feature Extraction Technologies

| Literature | Algorithm Architecture | Input Data Type | Feature Alignment Method | Effects | UPDRS Prediction Performance |
| --- | --- | --- | --- | --- | --- |
| Sigcha et al. | Multi-Task CNN | Consumer-Grade Smartwatch Triaxial Accelerometer | Time Window Segmentation (2.56 Seconds) | Tremor Detection Sensitivity 86.1%, Specificity 86.1%, AUC=0.936 | Tremor Amplitude Persistence Strongly Correlated with UPDRS Subitems |
| Borzì et al. | Machine Learning, Deep Learning, Single Threshold Method | Single Inertial Sensor on Waist | 2-Second Sliding Window | FoG Prediction Sensitivity 0.96, Specificity 0.93 | FoG Frequency Positively Correlated with UPDRS-III |
| Amato et al. | K-Nearest Neighbor (kNN) Classifier | Speech Signal (Isolated Word Pronunciation) | Range Normalization | PC-GITA Test Set Accuracy 94.3%, Sensitivity and Specificity Both Nearly 99% | Correlated MDS-UPDRS |
| Park et al. | Deep Neural Network (DNN), Recursive Feature Elimination | Hand Three-Axis IIMU Angular Velocity Signal | Periodic Integration Error Elimination | Patient Classification Performance AUC=0.926 | IMU Score Strongly Correlated with UPDRS-III (rho>0.84) |
| Pegolo et al. | Facial Landmark Tracking, K-Nearest Neighbor (kNN), Random Forest, Neural Network, Leave-One-Out Method | 2D Facial Video | Normalization of distance values ​​under neutral expressions, averaging of 4 consecutive frames, and removal of outliers using the interquartile range (IQR) | Distinguishing PD from Healthy People (HC), Excellent Performance in High Emotion Classification (AUC ≈ 88.9% for HC, 88.4% for PD) | FMI provides an independent and objective metric for assessing facial movement disorders |

Table 6. Cross-Modal Fusion Strategy Technology Comparison Table

| Literature | Algorithm Architecture | Input Data Type | Feature Alignment Method | Effects | UPDRS Prediction Performance |
| --- | --- | --- | --- | --- | --- |
| Skibińska et al. | XGBoost | Speech Signal, Facial Video | Task Segment Alignment | Balanced Accuracy 83%, Sensitivity 88%, Specificity 78% | Mouth movement features are correlated with UPDRS-III (p<0.05) |
| Moore et al. | YOLOv8, overlap detection, binary masking, and logical operations | Video, waist IMU gait data | Timestamp Synchronization, Path Overlap Detection, Object Category and Gait Parameter Correlation Analysis | Accurately detect objects in the environment (mAP50 = 0.81)  Improved Gait Interpretation, Effective Privacy Protection | Gait asymmetry and gaze obstruction neglect are associated with UPDRS gait items |
| Shin et al. | CNN-LSTM hybrid model | EMG signals, sound data | Swallowing Event Timestamp Alignment | Silent Aspiration Detection Specificity 0.99 | Swallowing duration can be indirectly mapped to UPDRS III swallowing items |
| Lipsmeier F et al. | Machine Learning, intraclass correlation coefficient (ICCs),Mann ‑ Whitney U test | Active test data (motion, speech, cognition, tremor) and passive test data (gait monitoring and non gait arm movement) | Time window aggregation, clinical data alignment, quality control | The test-retest reliability of all predefined sensor characteristics has a median ICC=0.9 and a range of 0.75 – 0.95. The sensor characteristics have the ability to distinguish mild symptoms, disease stages, and the severity of both sides of the body. The active test completion rate is 96.29%. | There was a statistically significant correlation with the total score of MDS ‑ UPDRS and sub scores (such as movement, posture, tremor) |

Table 7. Comparison of Federated Learning Application Technologies

| Literature | Algorithm Architecture | Input Data Type | Privacy Protection Mechanism | Effects | Clinical Implementation Challenges |
| --- | --- | --- | --- | --- | --- |
| Shen et al. | Federated learning, meta-learning combination, attention mechanism | Motion sensors, environmental sensors, smartphone status sensors, location information | No raw data transmission, FedAvg strategy aggregation update, user anonymization | DivAR Outperforms baseline models in both accuracy and F1 score | Requires a large amount of computing resources from edge devices |
| Gad et al. | Knowledge Distillation (KD), enhancement mechanism | Self-collected dataset (HARB): gyroscope and photoplethysmography sensors, public dataset (HARS): smartphone embedded inertial sensors | Sharing only soft labels or model output probabilities, knowledge distillation enhances privacy | The best performing model on the HARS dataset achieved 95.4%, and the best performing model on the HARB dataset achieved 67.8%, | The public dataset relied on should ideally have a distribution similar to that of patient data but an independent source |
